# Adhesion receptors as therapeutic targets for circulating tumor cells

**DOI:** 10.3389/fonc.2012.00079

**Published:** 2012-07-24

**Authors:** Jiahe Li, Michael R. King

**Affiliations:** Department of Biomedical Engineering, Cornell UniversityIthaca, NY, USA

**Keywords:** adhesion, receptors, CTCs, cancer therapy

## Abstract

Metastasis contributes to >90% of cancer-associated mortality. Though primary tumors can be removed by surgical resection or chemo/radiotherapy, metastatic disease is a great challenge to treatment due to its systemic nature. As metastatic “seeds,” circulating tumor cells (CTCs) are believed to be responsible for dissemination from a primary tumor to anatomically distant organs. Despite the possibility of physical trapping of CTCs in microvessels, recent advances have provided insights into the involvement of a variety of adhesion molecules on CTCs. Such adhesion molecules facilitate direct interaction with the endothelium in specific tissues or indirectly through leukocytes. Importantly, significant progress has been made in understanding how these receptors confer enhanced invasion and survival advantage during hematogenous circulation of CTCs through recruitment of macrophages, neutrophils, platelets, and other cells. This review highlights the identification of novel adhesion molecules and how blocking their function can compromise successful seeding and colonization of CTCs in new microenvironment. Encouraged by existing diagnostic tools to identify and isolate CTCs, strategic targeting of these adhesion molecules to deliver conventional chemotherapeutics or novel apoptotic signals is discussed for the neutralization of CTCs in the circulation.

## Introduction

Circulating tumor cells (CTCs) are cells that leave a primary tumor and circulate in the blood. More than a century ago the Australian physician Thomas Ashworth first observed CTCs in the blood of a patient with metastatic cancer. He hypothesized that “the cancer itself being seen in the blood may tend to throw some light upon the mode of origin of multiple tumors existing in the same person.” In the past decade, advancing technologies to detect and isolate CTCs have provided unique fluid biopsy information for prognosis, management of chemotherapy dosing and timing as well as monitoring the development of drug resistance over time (Nagrath et al., 2007; Lowes et al., 2011; Scher et al., 2011). Driven by these technologies, numerous clinical studies performed for breast, colon, prostate, and other epithelial cancers establish a clear connection between average CTC counts and overall survival rate before and during treatment (Cristofanilli et al., 2004, 2005; Hou et al., 2009; Criscitiello et al., 2010; Yalcin et al., 2010; Danila et al., 2011; Lianidou and Markou, 2011).

In contrast to the rapid development of tools for CTC detection and isolation, effective therapies that directly remove CTCs from the blood circulation are still underexplored. This is probably attributable to our limited understanding of the heterogeneity of CTCs: in most studies, CTCs are defined as being positive for epithelial cell adhesion molecule (EpCAM+) and cytokeratin 8, 18, or 19 (CK+) and negative for CD45 (CD45−) (Allard et al., 2004). However, almost a third of patients with advanced breast, colorectal, and prostate cancers have CTCs that do not meet these criteria (Coumans et al., 2010). Despite the heterogeneity of CTC markers, some studies have shown that cancer stem cell (CSC) or stem-like cell (CSC-like) markers are frequently expressed by CTCs (Aktas et al., 2009; Theodoropoulos et al., 2010; Iinuma et al., 2011; Toloudi et al., 2011; Kasimir-Bauer et al., 2012; Wang et al., 2012). Such features are especially relevant for targeting CTCs as CSCs are believed to represent a subpopulation of cancer cells that drive the growth and progression of metastatic cancers (Ghiaur et al., 2012; Vermeulen et al., 2012).

The presence of CTCs in the circulation can in part explain a clinical observation that the removal of a primary tumor is often followed with distant metastasis and/or local recurrence. For example, it was estimated that 20–50% patients first diagnosed with primary breast cancer eventually developed metastatic disease in the past (Lu et al., 2009). In the case of hepatocellular carcinoma (HCC), liver transplantation is the best treatment for early-stage patients. Unfortunately, every year around 10% of recipients develop post-transplant HCC recurrence, which leads to death in almost all patients (Toso et al., 2011). To understand the molecular mechanism, Kim and colleagues developed a tumor self-seeding mouse model whereby the local recurrence mediated by CTCs was investigated using human colorectal, melanoma, and breast cancer cells. They found that tumor-derived IL-6 and IL-8 served as CTC attractants whereas the seeder CTCs highly expressed invasion-associated genes (*MMP1*, *FSCN1*, and *CXCL1*) to promote infiltration (Kim et al., 2009). This finding highlights a highly orchestrated process of local recurrence mediated by CTCs.

**Figure 1:**
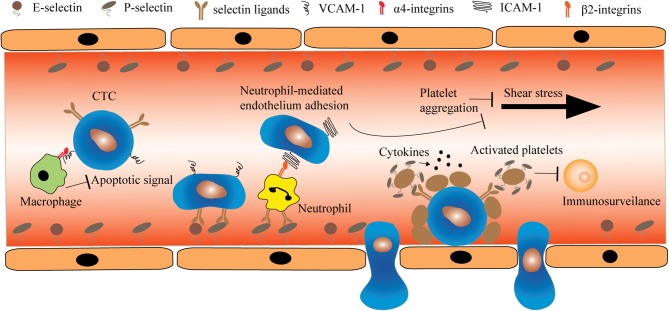
**The heterotypic cell interactions between CTCs and haemopotoeic cells in the circulation.** Macrophages interact with CTCs via α4-integrin-VCAM-1 ligation and transmit anti-apoptotic signal such as TRAIL to CTCs. In contrast, certain CTCs deficient for selectin ligands can roll and adhere to endothelial cells through heterotypic interaction with neutrophils. This interaction is mediated by β2-integrin and ICAM-1 expressed on neutrophils and CTCs, respectively. Additionally, CTCs can locally induce platelet aggregation. The deposition of platelets on CTCs prevents the damage caused by shear stress and immuno-surveillance.

CTCs play a predominant role in the metastases to distant organs. In the blood circulation, CTCs are subject to a multitude of stresses including anchorage-dependent survival signaling, immuno-surveilance, and shear stress. For example, CTCs are deprived of integrin-dependent adhesion to extracellular matrix (ECM) components in comparison to nontransformed cells. Whereas such loss of anchorage induces apoptosis (anoikis) in normal cell types, CTCs are particularly resistant to anoikis by promoting PI3K/Akt proliferation signaling and expression of anti-apoptotic proteins such as BCL2 (Frisch and Ruoslahti, 1997; Guo and Giancotti, 2004). Notably, numerous research has demonstrated that CTCs do not mobilize in the circulation alone. Instead, through heterotypic interactions with endothelial cells and different types of haemopotoeic cells, CTCs acquire the potential to metastasize to distant organs (Figure 1). Therefore, such receptor-mediated adhesion can provide a unique opportunity for neutralizing CTCs either through the blockade of receptors or receptor-targeted drug delivery.

## The biology of selectin-mediated hematogenous metastasis

Selectins are transmembrane glycoproteins which were initially found to bind specific glycoproteins on leukocytes. Three structurally related adhesion molecules, L-, E-, and P-selectin are composed of an N-terminal C-type lectin domain which confers specific, Ca^2+^-dependent carbohydrate-binding activity. It is followed by an epidermal growth factor (EGF)-like domain, a variable number of short consensus repeats domains (2, 6, and 9 for L-, E-, and P-selectin, respectively), a single-pass transmembrane domain and a short intracellular C-terminal tail (Ley, 2003). Despite structural similarity, the three selectins have distinct tissue-specific expression and binding kinetics. L-selectin is constitutively expressed on the surface of almost all types of leukocytes and is cleaved from the cell surface upon activation with a variety of cytokines and chemokines (Grailer et al., 2009). In contrast, the expression of E- and P-selectin is inducible on vascular endothelial cells during inflammation. Whereas E-selectin depends on de novo mRNA synthesis, P-selectin is stored in Weibel-Palade bodies of endothelial cells. Additionally, platelets express P-selectin which translocates from α-granules upon platelet activation (Larsen et al., 1992).

The role of selectins in mediating the rolling and trafficking of neutrophils and monocytes to inflammation sites has been well studied. More recently, it has been proposed that CTCs adopt similar strategies to facilitate their initial entrapment in the vessels and subsequent extravasation. Köhler and colleagues provided the first *in vivo* evidence that E- and P-selectin are essential for colorectal cancer metastasis. They generated a transgenic immuno-compromised mouse with E- and P-selectin doubly knocked out. Compared to wild-type mice, the double knockout mice with subcutaneously implanted colon cancer cells showed lung metastases reduced in number by 84% (Kohler et al., 2010). In agreement with earlier *in vitro* studies, a model was proposed in which the sialylated fucosylated glycans decorated on transmembrane proteins or specific lipids of CTCs mediate the rolling and adhesion to selectin-expressing endothelial cells. The role of selectin ligands in mediating the hematogenous metastasis of CTCs has been reviewed extensively elsewhere (Konstantopoulos and Thomas, 2009; Geng et al., 2012). However, this section focuses on therapeutic interventions of selectin binding that have been explored for the prevention of metastasis.

### Carbohydrate-based inhibitors

Given that all three selectins recognize sialylated fucosylated glycans such as sLe^x^, the sLe^x^ analogs have been shown to significantly prevent neutrophil accumulation and myocardial necrosis after ischemia and reperfusion in animal models (Buerke et al., 1994; Lefer et al., 1994; Zacharowski et al., 1999). This implies that the same analogs may be potent inhibitors for reducing CTC adhesion to endothelium. Shirota and colleagues investigated the inhibitory effect of a sLe^x^ analog, GSC-150 on hepatic metastasis of human colon carcinoma in nude mice. They found that liver metastases were significantly attenuated when cancer cells were co-administered with GSC-150 (Shirota et al., 2001). In addition to sLe^x^ analogs, novel disaccharides have been generated which function as competitive substrate inhibitors for glycotransferases involved in the synthesis of sLe^x^. To this end, a disaccharide compound was able to inhibit sLe^x^ formation in human monocytic leukemia cells, U937. Its therapeutic effect was further studied in Lewis lung carcinoma *in vivo* where the experimental metastasis was significantly reduced through the decreased expression of sLe^x^ (Brown et al., 2009). Nevertheless, strategies to abrogate sLe^x^-selectin interaction must be considered carefully. Given the turnover rate of selectins or glycotransferases, such carbohydrates may not have a long-lasting inhibitory effect. Moreover, as sLe^x^ is essential for directing neutrophils and lymphocytes to inflamed tissues, the chronic exposure to sLe^x^ analogs or metabolic inhibitors can interfere with the normal inflammatory response. Therefore, investigations on the cellular sLe^x^ synthesis that differentiate CTCs from leukocytes may provide more specific targeting of CTCs while reducing side effects.

### Gene silencing of fucosyltransferases in CTCs

As the key determinants of selectin ligands, sLe^x^ and sLe^a^ are synthesized in the Golgi compartments by sequential actions of N-acetylglucosaminyl-, galactosyl-, sialyl-, and fucosyl-transferases. Of note is the terminal step of transferring fucose to N-acetylglucosamine catalyzed by a family of fucosyltransferase genes (Hennet, 2002). At least nine *FUT* genes have been identified in the human genome among which *FUT*3, 4, 6, and 7 have been well characterized. They are redundant in the synthesis of sialyl lewis carbohydrates but display cell type-specific expression. FUT4 and FUT7 are mainly expressed in blood cell lineages and play a key role in the selectin ligand-mediated migration of leukocytes during the inflammatory response (Weninger et al., 2000). In contrast, FUT3 and FUT6 are more associated with the progression of cancers, including breast (Matsuura et al., 1998; Ding and Zheng, 2004), prostate (Barthel et al., 2008), lung (Ogawa et al., 1996), liver (Wang et al., 2003), and gastric cancer (Petretti et al., 1999). To exploit the therapeutic potential of targeting fucosyltransferases, our laboratory first confirmed that hematopoietic cell lines (HL60 and KG1a) predominantly express FUT4 and FUT7 whereas prostate cancer cell line MDA PCa2b mainly expresses FUT3. Next, siRNA against FUT3 reduced sLe^x^ expression on prostate cancer cells and significantly inhibited cell rolling and adhesion to a E-selectin-functionalized surface under physiological flow. In addition, the siRNA was able to impair cell growth which may not be directly associated with sLe^x^. In fact, two recent studies revealed that the overexpression of FUT4 and FUT6 promoted cell growth by elevating intracellular Akt phosphorylation and suppressing the cyclin-dependent kinase inhibitor p21 in epidermoid carcinoma and HCC cells, respectively, (Yang et al., 2010; Guo et al., 2012). Therefore, silencing FUTs via siRNA can simultaneously inhibit the adhesion and clonal expansion of CTCs in the blood circulation. To apply this strategy *in vivo*, P-selectin-based liposome nanoparticles recently developed in our laboratory can be used to encapsulate siRNAs against FUTs (Huang and King, 2009). Although P-selectin recognizes both circulating leukocytes and CTCs, siRNAs against FUTs exclusively expressed in CTCs provide additional targeting specificity.

## Therapeutic abrogation of CTC-hematopoietic cell interaction

It is estimated that less than 0.01% of CTCs shed from a primary tumor can survive to produce clinically relevant metastases (Joyce and Pollard, 2009). This suggests that the process of metastasis by CTCs is largely inefficient. Whereas the mechanisms underlying such high rates of attrition remain poorly understood, recent studies identified two important cell adhesion molecules involved in the physical interactions of CTCs with hematopoietic cells: vascular cell adhesion molecule-1 (VCAM-1) and intercellular adhesion molecule-1 (ICAM-1). Such interactions facilitate CTCs in several aspects: (1) survival in the circulation, (2) initial arrest and subsequent extravasation, and (3) eventual growth into overt metastasis (Chambers et al., 2002).

The transmembrane protein VCAM-1 was originally thought to be presented exclusively on endothelial cells in response to tumor necrosis factor-alpha (TNF-α) and interleukin-1 (IL-1) during inflammation (Coussens and Werb, 2002). It binds to the leukocyte integrins α4β1 and α4β7 on circulating monocytes, granulocytes, and lymphocytes (Osborn et al., 1989; Elices et al., 1990). However, aberrant expression of VCAM-1 was found to be one of 18 signature genes associated with lung metastasis of breast cancer in both experimental mouse models and patients (Minn et al., 2005). Chen and colleagues found that VCAM-1 on breast cancer CTCs tethered to metastasis-associated macrophages which express α4-integrins. Clustering of VCAM-1 on CTCs induces Akt activation and protects CTCs from proapoptotic cytokines such as TNF-related apoptosis-inducing ligand (TRAIL). Notably, either silencing VCAM-1 expression by siRNA or blocking antibody against α4-integrins abolished the pro-survival effect of VCAM-1 (Chen et al., 2011). In addition, VCAM-1 was also recently found to be associated with bone metastasis in breast cancer. Prior to this study, a bone-metastatic gene signature including *CXCR4*, *IL11*, *CTGF*, *MMP1*, and *OPN* was identified through the reiterative selection of human breast cancer cells MDA-MB-231 in immuno-compromised mice (Kang et al., 2003). However, by studying a subpopulation of MDA-MB-231 which experienced a long dormancy prior to bone metastasis, Lu and colleagues discovered that the aberrant expression of VCAM-1 engaged α4-integrins on monocytic osteoclast progenitors to promote the local osteolytic activity in bone (Lu et al., 2011). This mouse study was further corroborated by comparing VCAM-1 levels between clinical early and late recurrences of bone metastases. Higher VCAM-1 was significantly associated with early relapse (Wang et al., 2005).

Like VCAM-1, ICAM-1 is another cell surface glycoprotein which is typically expressed on endothelial cells in response to TNF-α or IL-1 in inflammation. However, the constitutive expression of ICAM-1 on CTCs was found to promote tumor cell transendothelial migration in melanoma (Huh et al., 2010), pancreatic (Roland et al., 2010), and breast cancers (Wu et al., 2001). To understand this mechanism, *in vitro* biophysical studies demonstrated that under physiological shear stress ICAM-1 on melanoma CTCs promotes the heterotypic interaction with neutrophils by engaging β2-integrins (CD11a and CD11b) (Hoskins and Dong, 2006; Liang et al., 2008). Moreover, as neutrophils have selectin ligands, such heterotypic interaction facilitates the adhesion and extravasation of melanoma CTCs which otherwise bind inefficiently to the endothelium (Slattery and Dong, 2003; Liang et al., 2005). Later, our laboratory studied the physical mechanisms of retinoblastoma metastasis. Whereas human RB cell lines RB143 and WERI-Rb27 do not express E-selectin ligands, they can be recruited to an E-selectin-coated surface through attachment to activated neutrophils. This interaction is also mediated by ICAM-1: β2-integrin (Geng et al., 2010). To test the involvement of this heterotypic interaction *in vivo*, Jin Huh and colleagues compared the lung metastases of human melanoma cells injected alone or in combination with human neutrophils (Huh et al., 2010). They found that human neutrophils enhanced CTCs retention in the lung by three-fold. To dissect the molecular mechanism, the cytokine interleukin-8 (IL-8) was found to be a key determinant expressed by melanoma cells to attract neutrophils. IL-8 secretion increased β2-integrin levels on neutrophils and heterotypic aggregation between ICAM-1-positive CTCs and neutrophils. Importantly, siRNA against IL-8 impaired transendothelial migration and lung metastasis by ~50%. In addition to targeting IL-8 as a therapeutic approach, it is possible that blocking antibodies against ICAM-1 or β2-integrins may be also effective (Rosette et al., 2005).

## Platelets aggravate CTC metastasis

Platelets are anuclear cytoplasmic bodies released from megakaryocytes in the bone marrow. It is estimated that one liter of blood contains about 400 billion circulating platelets. The primary role of platelets is to maintain haemostasis. This is initiated via platelet activation which results in adhesion and release of a multitude of bioactive factors from platelet granules. In addition to haemostatic regulation, platelets have long been believed to play a critical role in cancer metastasis through the enhancement of CTC survival and adhesion to the endothelium in the circulation. The involvement of platelets in cancer was first recorded in the mid-nineteenth century by the French clinician Armand Trousseau. He diagnosed patients with migratory thrombophlebitis caused by an occult visceral carcinoma (Gupta and Massague, 2004). In fact, preclinical studies in genetic knock-out mice provide evidence that upon immediate entry into the circulation, tissue factor highly expressed by CTCs can signal downstream through FVIIa and FXa to activate a coagulation cascade leading to thrombin generation, fibrin deposition, and platelet aggregation around CTCs (Camerer et al., 2004; Kasthuri et al., 2009; Liu et al., 2011). Such a “platelet cloak” is known to initially trap tumor cells in microvessels (Borsig et al., 2001).

Several mechanisms of platelets in promoting CTC survival have been proposed based on preclinical experiments in mice using a variety of mouse and human carcinoma cell lines. Aggregation of platelets around CTCs protect against immune-mediated clearance of CTCs largely mediated by natural killer (NK) cells. The potential of CTCs to induce platelet aggregation correlates with their enhanced metastatic potential. Bernhard Nieswandt and colleagues demonstrated for the first time that platelets directly impair NK lysis of tumor cells *in vitro* and *in vivo*. In a mouse model of experimental metastasis, they found that tumor seeding in the lung was reduced when platelets were depleted from the host (Nieswandt et al., 1999). Further studies reveal that CTC evasion of NK cells is not merely attributed to physical shielding of platelets. NK cell activity is guided by the principles of “missing-self” and “induced-self,” which imply that cells lacking expression of MHC class I (missing-self) and/or a stress-induced expression of ligands for activating NK receptors (induced-self) are preferentially recognized and eliminated (Moretta and Moretta, 2004; Lanier, 2005). While CTCs are often associated with lack of MHC class I, platelets can disrupt “missing self” recognition of NK cells by grafting MHC I class onto CTCs (Placke et al., 2012). Furthermore, platelet-derived transforming growth factor β (TGF-β) can downregualte the activating immunoreceptor NK group 2, member D (NKG2D) on NK cells (Kopp et al., 2009).

### Therapeutic intervention of platelet adhesion to CTCs

As the blood clotting pathway contributes to platelet adhesion to CTCs, a variety of anticoagulation agents have been tested either alone or together with conventional cancer drugs in preclinical mouse models. Using an experimental metastasis mouse model, Amirkhosravi and colleagues found that the intravenous injection of recombinant mouse tissue factor pathway inhibitor (TFPI) immediately before inoculation of tumor cells reduced metastasis by 83% (Amirkhosravi et al., 2002, 2007). Similarly, Cilostazol, a selective inhibitor of phosphodiesterase 3 with anticoagulatory and profibrinolytic effects completely abolished the complex formation of 4T1 tumor cells in the presence of activated platelets *in vitro*. In a spontaneous model of mouse 4T1 breast cancer, the injection of Cilostazol six hours before tumor inoculation reduced pulmonary metastasis by 55%. As platelet aggregation and adhesion to CTCs enhance their survival in the blood circulation, abrogation of the coagulation cascade renders CTCs susceptible to cancer drugs. Wenzel and colleagues invented dual liposomes simultaneously containing the hemostatic inhibitor dipyridamole and the anticancer drug perifosine. The liposomes caused a 90% reduction in the number of lung metastases in a mouse experimental metastasis model (Wenzel et al., 2010).

Despite the fact that anticoagulants hold promise for the prevention of metastasis, they may impair the normal hemostatic function of platelets in the presence of bleeding. Platelet intervention therapies against metastasis must exhibit certain specificity for tumor cell-platelet interactions. Therefore, direct inhibition of platelet adhesion to CTCs may minimize the cardiovascular side effect of anticoagulants. To this goal, heparin and chemically modified heparins have been shown to attenuate the metastasis of human colon carcinoma in a mouse xenograft model (Koenig et al., 1998; Stevenson et al., 2005; Hostettler et al., 2007). The anti-metastatic effect of heparin was initially believed to associate with its anticoagulant activity. Later it was found that competitive binding of heparin to P-selectin on activated platelets abolishes interaction with P-selectin ligands such as sialylated fucosylated mucins expressed on human colon carcinoma cells (Wei et al., 2004; Stevenson et al., 2005; Hostettler et al., 2007; Lee et al., 2008). As the anticoagulant activity of heparin is undesirable in the context of blocking CTC-platelet interactions, polysaccharides isolated from certain sea plants and fungi have shown enhanced inhibition of P-selectin binding without anticoagulant effect. A fucosylated chondroitin sulfate (FucCS) from sea cucumber is 4–8 fold more potent than heparin in the inhibition of LS180 carcinoma cell attachment to immobilized P- and L-selectin. Moreover, administration of FucCS 30 min prior to mouse colon carcinoma MC-38 injection is associated with 2-fold CTC-platelet aggregates than heparin in the mouse lung. Long-term experiment reveals that FuCS significantly reduced lung metastatic foci by 80% compared to saline control (Borsig et al., 2007).

### Therapeutic blockage of platelet signaling to CTCs

Certain CTCs express epithelial markers such as EpCAM and cytokeratins, suggesting that the epithelial-mesenchymal transition (EMT) is not necessarily required for CTCs to access the blood circulation. Instead, a transient contact between platelets and CTCs in the blood circulation is sufficient to induce an EMT gene signature and invasive behavior primarily through the platelet-secreted transforming growth factor-β1 (TGF-β1) (Labelle et al., 2011). Recently, a small molecule inhibitor, SD-208, has been shown to block the TGF-β receptor I kinase (TβRI) activity. SD-208 successfully prevented the development of TGF-β-induced bone metastases and decreased the progression of established osteolytic lesions in a melanoma mouse model (Mohammad et al., 2011). Therefore, SD-208 possibly represents a viable therapeutic to inhibit platelet-derived TGF-β signaling. In addition to TGF-β, platelet α-granules store abundant proangiogenic factors including vascular endothelial growth factor (VEGF), basic fibroblast growth factor (bFGF), EGF, platelet-derived growth factor (PDGF) and insulin-like growth factor-1 and -2 (IGF-1 and -2) (Sierko and Wojtukiewicz, 2007). Given that inhibitors for the proangiogenic factors or their counter-receptors are available as cancer drugs in the treatment of solid tumors (Roberts et al., 2005; Moreira et al., 2007; Weroha and Haluska, 2008), it is possible that such inhibitors can be used as adjuvant therapies in the context of targeting CTCs.

## Novel selectin-based targeting drug delivery to CTCs

Over the past several years, our laboratory has developed a biomimetic approach to isolate CTCs using a selectin-immobilized microtube device (Hughes and King, 2010; Hughes et al., 2012a,b). Two factors are responsible for the efficient capture of CTCs by this device. First, the ability of selectins to mediate the rapid tethering and rolling of leukocytes or CTCs under shear is attributed to the fast kinetics between selectins and selectin ligands (Lawrence and Springer, 1991; Wild et al., 2001). The fact that cells can be enriched under flow conditions significantly enhances the sample processing rate. Second, the microtube allows for the margination of CTCs toward the wall to interact with immobilized selectins. This margination effect has been well characterized when leukocytes circulate in a flow-dependent interaction with red blood cells (Bagge et al., 1983; Goldsmith and Spain, 1984; Iadocicco et al., 2002).

**Figure 2:**
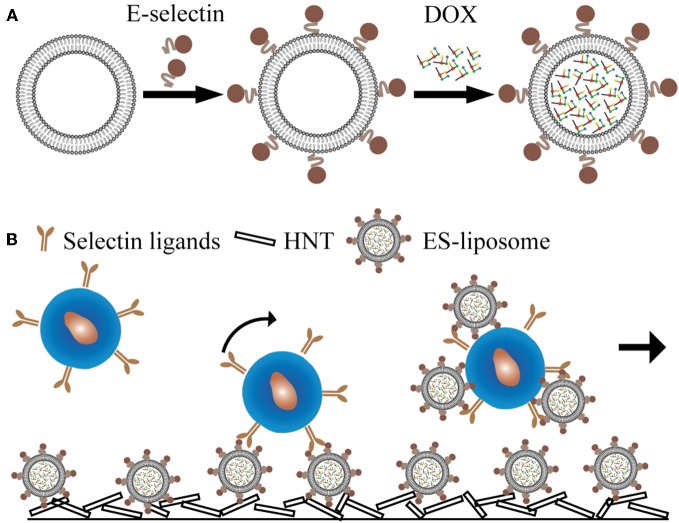
**E-selectin liposomal and nanotube-targeted delivery of doxorubicin to CTCs. (A)** Schematic of E-selectin-coated liposome encapsulating doxorubicin (DOX). **(B)** Schematic of a microtube device for delivering DOX to captured CTCs.

Inspired by isolating CTCs under flow conditions, we translated the device to a unique drug delivery platform whereby the immobilization of drug molecules on the surface creates a high localized concentration. One device immobilizes E-selectin-conjugated liposomes onto the surface of a blood-compatible microrenathane (MRE) tube. After encapsulating doxorubicin (DOX), the liposomes could specifically capture cells from the flow and efficiently deliver DOX into adherent cells. Moreover, a halloysite nanotube (HNT)-coated surface further enhanced the targeting and killing of cancer cells (Figures 2A,B), which was attributed to the increased surface area for both E-selectin and DOX.

**Figure 3:**
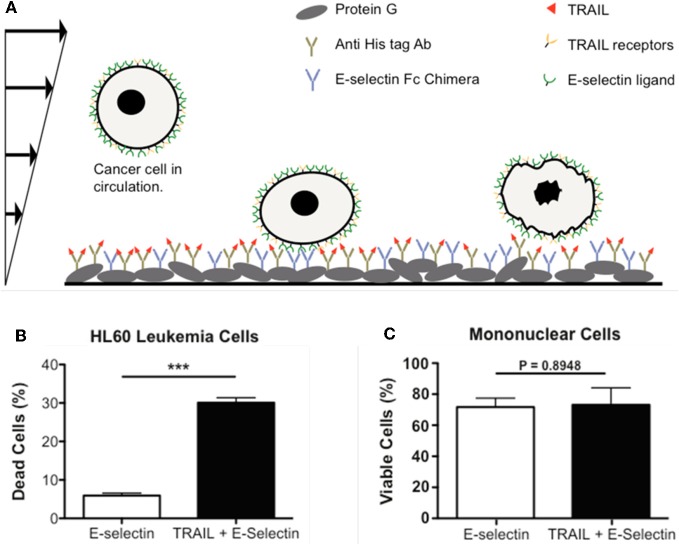
**Delivery of apoptotic signal to rolling cancer cells. (A)** Schematic of a biomimetic device for inducing apoptosis in CTCs using E-selectin and TRAIL. **(B)** HL60 leukemic cells are eliminated by approximately 30% following perfusion through a device coated with both E-selectin and TRAIL. **(C)** Viability of normal mononuclear cells is not affected by the TRAIL-coating, confirming the specificity of TRAIL for malignant cells. **(A–C)** are reproduced from Rana et al., 2009 with permission. Rana et al., 2009 is Copyright 2008 Wiley Periodicals, Inc.).

To provide more specificity to CTCs, two additional approaches have been pursued by our laboratory. One approach was to functionalize the microtube surface with both E-selectin and antibodies against epithelial markers such as EpCAM. Such additional antibodies were able to discriminate between leukocytes and CTCs when cells roll on the surface (Hughes et al., 2012a). The second approach was to replace DOX with molecules that are tumor-specific, such as TRAIL. TRAIL holds promise as a tumor-specific therapeutic as it selectively induces an apoptotic signal by binding to death receptors on the cell surface (Koschny et al., 2007; Wang, 2008). To this end, our lab developed a death receptor-mediated apoptosis device to deliver apoptosis signal to captured CTCs (Figure 3A). Notably, with TRAIL and E-selectin on the surface, one hour of rolling exposure was sufficient to kill 30% of leukemia cells (HL60) whereas the viability of normal mononuclear cells was not affected (Figures 3B,C) (Rana et al., 2009, 2012)

## Concluding remarks

In haematogenous metastasis, a primary tumor sheds CTCs into the blood circulation which comprise a population of carcinoma cells that can exhibit CSC or CSC-like features. Two hypotheses have been proposed regarding how CTCs establish the initial contact with endothelial cells prior to metastasis. The physical trapping hypothesis is based on the fact that the luminal diameter of capillaries is ~8 μm while the diameter of CTCs ranges from 20 to 30 μm. Thus, CTCs can be simply mechanically trapped in the capillary bed during their first pass through the circulation (Valastyan and Weinberg, 2011). However, the findings that certain CTCs display organ-specific tropism (e.g., bone metastases in breast and prostate cancer) challenge this hypothesis (Kang et al., 2003; Barthel et al., 2008). In fact, by displaying selectin ligands on their surface, CTCs in certain cancers acquire the ability to roll and adhere to the endothelium and subsequently exit from the circulation. The identification of selectin-dependent metastasis has made it possible to develop a range of antagonists against selectins or selectin ligands. Such antagonists have proven efficient in reducing experimental metastases in many mouse cancer models. However, they may impair the selectin-dependent trafficking of leukocytes to inflamed areas during the normal inflammatory response. The gene silencing of specific FUTs may confer specificity to CTCs as different FUTs have been shown to differentially express in CTCs versus leukocytes (Yin et al., 2010). Nevertheless, the CTC-endothelium interaction alone is not sufficient for CTCs to overcome damages incurred by hemodynamic shear forces and immuno-surveillance. The survival of CTCs in the blood circulation also depends on the interactions with haemopoetic cells such as macrophages, neutrophils, and platelets which require distinct adhesion receptors. Though the abrogation of individual adhesion receptors has shown promising results in a variety of mouse cancer models, it may be clinically relevant to develop a cocktail therapy which simultaneously targets multiple interactions between CTCs and other cell types.

### Conflict of interest statement

Michael R. King is a scientific advisor of CellTraffix, Inc. Jiahe Li has no commercial or financial relationships that could be construed as a potential conflict of interest.
